# What Explains Natives and Sojourners Preventive Health Behavior in a Pandemic: Role of Media and Scientific Self-Efficacy

**DOI:** 10.3389/fpsyg.2021.664399

**Published:** 2021-06-29

**Authors:** Fang Keren, Ahmad Nabeel Siddiquei, Muhammad Azfar Anwar, Fahad Asmi, Qing Ye

**Affiliations:** ^1^School of Journalism and Communication, Anhui University, Hefei, China; ^2^Bond Business School, Bond University, Gold Coast, QLD, Australia; ^3^School of Management, University of Shenzhen, Shenzhen, China; ^4^Department of Science and Technology of Communication, University of Science and Technology of China, Hefei, China; ^5^Key Laboratory of Immersive Media Technology (Anhui Xinhua Media Co, Ltd.), Ministry of Culture and Tourism, Hefei, China

**Keywords:** health belief model, media self-efficacy, scientific self-efficacy, China, sojourners, COVID-19

## Abstract

The COVID-19 pandemic triggered a severe global public health emergency. The current research investigated and compared “Natives and Sojourners” health-protective behavior in Mainland China during the pandemic. We adopted a unified view to propose our theoretical model by adapting the Health Belief Model (HBM) and Institutional Theory (IT). The data obtained through an online survey questionnaire from 435 respondents during the second and third quarters of were analyzed. Structural equation modeling (SEM) was used to empirically analyze the proposed model. The media self-efficacy (MSE), scientific self-efficacy (SSE), perceived health risks (PHRs), and the perceived benefits of being protected have positive and significant effects on the definition of health-protective behavioral intentions among natives and sojourners in mainland China. Media and SSE can play a strategic role in formulating public health-protective behavior. The current research recommends an effective communication with sojourners during crisis for them to be a part of the national crisis management plan (i.e., infectious disease).

## Introduction

COVID-19 emerged as a pandemic in the first quarter of the year 2020 and had social (Yezli and Khan, 2020), ecological (Muhammad et al., 2020), political (Kerr et al., 2021), and economic (Qian et al., 2021) effects all over the world. At present, no internationally recognized measures are used as standards to cure COVID-19 patients (Li et al., 2020). However, preventive measures, e.g., wearing masks and gloves (Shahnazi et al., 2020), frequent washing hands (alcohol-based disinfectants or soap; Wang et al., 2021), restricting travel (Pandita et al., 2021), and physical distancing (Yezli and Khan, 2020), are implemented by institutions and governing bodies, because these measures are effective and helpful in controlling COVID-19 transmission. Although these all safety measures as discussed above have significant scientific justification, none of the existing research accounted for the role of scientific reasoning or individuals’ scientific self-efficacy (SSE) to map quantitative behavioral map in society.

In terms of health-protective measure during the current pandemic crisis, several constructive initiatives are observed in the developing (Yezli and Khan, 2020; Zandifar and Badrfam, 2020; Wang et al., 2021) and developed (Gallacher and Hossain, 2020; Heald et al., 2020; Kuchenbuch et al., 2020) countries. In China, strict measures are implemented and have helped normalize the situation. These measures included a nationwide lockdown (Fanelli and Piazza, 2020). All the provincial-level regions in Mainland China launched a top-level response to deal with the COVID-19 crisis in the first month of 2020, which included extending the Spring Festival holiday, postponing all school openings, and implementing strict travel restrictions (Xinhua, 2020). Though, the existing behavioral studies addressing COVID-19 have adapted several theoretical stances to enrich the understanding and process of conceptualizing health-protective behavior of individuals. The role of institutions and society as macro-level forces has not been recorded yet, especially in case of its moderating effect on the health-protective behavioral intentions.

At the time of the spread, 492,185 overseas students from 196 countries (Zhong, 2019) and almost one million expats were in China (People’s Daily, 2019). Sojourners are expats who usually face social integration issues in foreign countries. However, few researchers have investigated the psychological impact of the pandemic on sojourners, particularly how these sojourners acquire correct news (information) or what influences their health-protective behavior. As a significant difference exists among sojourners and natives, no trace of comparative analysis of sojourners and natives exists. Thus, a socio-psychological persuasive psychological model is needed to compare the health-protective behavior of sojourners and natives.

The COVID-19 outbreak has led to fear, worry, and anxiety worldwide. Shigemura et al. (2020) highlighted that COVID-19 has negatively impacted well-being, which aggravated the public’s fear and anxiety. Ma and Yan (2020) highlighted that the spread of false information of COVID-19 caused various kinds of fear, anxiety, and depression, which aggravated the difficulty of epidemic prevention and control. Zandifar and Badrfam (2020) also confirmed that misinformation about COVID-19 and social isolation have led to the public’s mental morbidity. Several studies stated that the Chinese and international media have a significant impact on the psychological response, perception, and knowledge-seeking behavior (Xie et al., 2020). Moreover, Gohel et al. (2021) stated that insufficient knowledge and lack of education are great challenges to surmount in dealing with the current pandemic crisis. The above-discussed trends and significant role of international and national media highlight that the cognitive factors of individuals need immediate need to underline individual’s ability to process and identify and access reliable sources of information. In other words, the literature signifies the role of media self-efficacy (MSE) and highlights the dire need to explore it further, especially in the time of crisis.

To address the research gaps as stated previously, this current research initiative aims to determine the role of media and SSE in formulating public health-protective behavior during a crisis. The current study adopted the theoretical perspective of cognitive psychology. The health concern factor (i.e., perceived exposure) was considered as exogenous to define individuals’ cognitive process and expectancy value-driven factors. Specifically, in the case of health concerns, the significant constructs defining avoidance (perceived benefits and barriers of being protected) and severity of the risk [perceived health risk (PHR)] were proposed in mapping health-protective behavior. The current research adopted the Rosenstock et al. (1988) stance, in which the fundamental attribute of self-efficacy from social learning theory was incorporated, diffused into Health Belief Model (HBM), and stretched further by introducing new streams of self-efficacies (media and scientific) in the HBM’s setting. During the current pandemic crisis, the roles of the normative environment (Cheng et al., 2020) and regulatory force (Al-Sabbagh et al., 2021) have been studied. However, the literature does not state where external (regulatory) and normative support can be recorded in a unified manner while adopting HBM. Therefore, to strengthen the novelty and to address the research gap, the Urban and Kujinga’s (2017) institutional view was adopted; in this view, only the regulative and normative environment is considered.

The current research had three significant contributions. First, the current study extended the spectrum of self-efficacies by discussing media and SSE simultaneously to present their strategic roles in perceived health-protective behavior in mainland China. Second, the current research incorporated the Urban and Kujinga’s (2017) institutional view and Rosenstock’s HBM to underline the role of normative and regulative environment in conceptualizing health-protective behavior. Moreover, the study compared the role of proposed efficacies (media and scientific) and institutional factors (regulatory and normative) in the case of sojourners and natives in mainland China.

## Theoretical Background

In the current century, the world has witnessed several health crises, i.e., Ebola, MERS, SARS, and COVID-19 (Laato et al., 2020); several research initiatives can be seen in the literature while emphasizing consumer behavior and risk mitigation or adopting strategies (La-Torre et al., 2009; Choi et al., 2017; Nie et al., 2020). Within the hood of health psychology, various behavioral theories exist. These theories underline the role of persuasive socio-psychological constructs while conceptualizing health-concerned behavior of individuals, e.g., conceptualizing the health-protective behavior of the workforce (Barello et al., 2020; Mathai, 2020; Almazyad et al., 2021) or being vaccinated to avoid serious health crisis (Hu et al., 2017; Zampetakis and Melas, 2021). In each scenario, most of the health-protective theories underline the constructs that can help mitigate the health challenges or adopt measures to avoid health challenges (i.e., pandemic crisis).

HBM is one of the most valuable frameworks for explaining health-related behavior, as it comprises the attributes of risk perception and behavioral evaluation (Cao et al., 2014). In the pre-COVID-19 literature, HBM has been used to underline the breast self-examination behavior (Didarloo et al., 2017), oral cancer prevention (Jeihooni et al., 2019), exercise-related injury prevention program participation (Gabriel et al., 2019), and healthy housing material selection of boomers (Kwon and Ahn, 2019). Moreover, during the current pandemic crisis, HBM has been used to determine the consumers’ perception of forest therapy tourism (Zhao and An, 2021), individual readiness towards home quarantine (Al-Sabbagh et al., 2021), the role of community pharmacists in offering effective communication to society (Carico et al., 2020), and citizens’ readiness to get vaccinated (Zampetakis and Melas, 2021). The intensive use of HBM in recent times signifies its significance and meaningfulness in depicting public health-protective behavior. Therefore, HBM was adopted in the current research initiative, because it helps define the following inter-construct relationships, including (1) the role of perceived sense of exposure in driving individuals’ preventive or protective behavior (Jones et al., 2015); (2) the current study further stretched the stance of Nexøe et al. (1999) as exogenous constructs (perceived barriers and benefits of being protected) in the HBM, which helped explain health-protective behavior. A similar pattern was observed in the study of Tong et al. (2020), who examined the attitude towards adopting precautionary measures during the current pandemic (COVID-19). Champion and Skinner (2008) reported that the fundamental constructs of HBM do not have definite relationships. To maximize the explanatory power of the research, several studies extended HBM (Zampetakis and Melas, 2021) or adopted other theoretical stances to incorporate with HBM, i.e., Theory of Planned Behavior (Zhao and An, 2021), Multidimensional Locus of Control Theory (Nexøe et al., 1999), and Social Cognitive Theory (Reid and Aiken, 2011). Jones et al. (2015) also analyzed different sets of arrangement within HBM, i.e., the parallel and serial mediating effects of benefits and barriers of being protected, to define health-protective behavior.

Carpenter (2010) observed that in the early traces of HBM, the essence of self-efficacy was rarely used. However, in recent decades, it became an integral part of HBM, as it holds a significant explanatory power to define public health concerns and protective behavior (Carico et al., 2020; Mirzaei-Alavijeh et al., 2020; Shahnazi et al., 2020; Tajeri et al., 2020). In existing HBM-based studies, self-efficacy was noted as a significant construct in exploring COVID19-related behavioral research (Niu et al., 2021). Zhou et al. (2020) suggested that correct news (information) could be an effective measure to control and reduce disease spread during the pandemic crisis, as self-efficacy enables individuals to seek valid and reliable health-related information in a more systematic fashion (Shang and Zuo, 2020). In particular, during COVID-19, the conspiracy theory endorsement was recorded as a great challenge, especially when considering international media sources in China; it signified the role of cognition (Su et al., 2021). In line with the arguments by Su et al. (2021) and Rosenstock et al. (1988), the current research bifurcated cognition to propose the media and SSEs, thereby helping explain how these efficacies can benefit information processing (Xiao et al., 2021) and allow individuals to think critically (Austin et al., 2016) while formulating perception of risk and health-protective behavior. Several researchers explained the role of media (Cheng et al., 2020) and scientific (Stosic et al., 2021) as a part of cognition (belief and efficacy) in the recent literature addressing COVID19-related behavioral studies.

To enrich the theoretical and practical novelty of the current research, the Institutional Theory (IT) was adopted to quantify the impact of endogenous factors as moderators. This theory has been used to study organizations and individuals within organizations (Kurtulmuş, 2019). Institutions can be understood as “rules of the game in a society” (North, 1990) that help shape individuals’ beliefs and their nonrational behaviors (Scott, 2001). The IT comprises three principal pillars, namely (1) regulative (2) normative, and (3) cognitive support (Scott, 2001). Urban and Kujinga (2017) stated that the regulative pillar refers to authorities’ regulations or guidelines to reward or punish actions (Valdez and Richardson, 2013). Normative pillar means the social norms of society that individuals attempt to comply with (Seelos et al., 2011). Cognitive pillar comprises the templates and scripts shared among a community or nation (Seelos et al., 2011). Wu et al. (2019) indicated that an individual’s intention and behavior are significantly affected by the normative environment. In particular, collectivist cultural countries have a high level of norms for individual behavior (Furnham et al., 2012; Shi et al., 2017). Thus, in the context of the current research setting, the proposed model took the regulative and normative concerns into account. These concerns can be diffused with HBM as cues to action, because HBM-related literature argues that cues can range from internal or external sources; cues to define health-protective behavior are still an underdeveloped attribute of HBM (Jones et al., 2015). Therefore, the current study takes this stance as an opportunity to use regulatory and normative support as a set of cues to act towards health-protective behavior. Bavel et al. (2020) argued that regulatory and social factors are essential to drive constructive social change and individual behavior during COVID-19.

Apart from the argumentation about the theoretical stance for the current research, the fundamental characteristics of sojourners and natives need to be studied and distinguished. Ang et al. (2017) indicated that migrant workers who have more financial barriers to obtain healthcare services are more likely to suffer from low psychological well-being. Dias et al. (2013) state that migrants experience numerous socio-cultural, legal, economic, and communicational constraints, which expose them to many health risks. For instance, immigrants from non-native language-speaking countries encounter linguistic barriers to healthcare and access to service. Furthermore, recent literature emphasized the impact of the source of information (news) in China when defining readers’ conceptualization of risk and fear of COVID-19 (Su et al., 2021); this source helps in the formulation of health orientation (Liu, 2021), drives negative emotion, and affects psychological resilience (Giri and Maurya, 2021). However, no previous report stated the use of the HBM in any setting to compare the sojourners and natives and to conceptualize the differences that can potentially drive practical implications and future research direction. Thus, in the context of the above-discussed argumentation, the following constructs’ relationships are proposed for sojourners and natives in Mainland China during the pandemic crisis to map their health-protective behavior.

## Hypotheses Development

Several recent studies emphasized the significance of HBM in examining the public’s behavior during the pandemic crisis (Almazyad et al., 2021; Al-Sabbagh et al., 2021; Hong et al., 2021; Wu et al., 2021). In the current research, the four constructs from the HBM, namely (1) perceived exposure (2) PHRs (3) benefits, and (4) barriers of being protected, were considered as exogenous to conceptualize health-protective behavior (Cao et al., 2014). Moreover, the proposed model also hypothesized the role of self-efficacy, which is an integral part of HBM, as suggested by Champion and Skinner (2008) and Jones et al. (2015) who proposed that media and SSEs are novel contributions. The study showed the role of Urban and Kujinga’s (2017) view of regulatory and normative support as cues to act in the setting of HBM. Within the HBM, the current study stretched the spectrum of self-efficacies and proposed a new view to examine the cues to act, because both of these attributes need more exploration to maximize the explanatory power of the HBM, as discussed by Jones et al. (2015).

In the view of HBM, perceived exposure (Exp) to any health issue can be considered as the trigger of human cognition and behavioral change (Rosenstock, 1974). Tajeri et al. (2020) stated perceived exposure as the degree of sensitivity to the situation, where the higher chances of being exposed push individuals to conceptualize the health risk. Zampetakis and Melas (2021) mentioned PHR as an individual’s perceived potential adverse consequences if the protective measures are ignored (Zampetakis and Melas, 2021). In the case of COVID-19, HBM implies that the higher sense of exposure and a greater degree of perceived risk can lead to compromising circumstances for the psychological well-being (i.e., anxiety and depression) if the coping strategies (i.e., social support) are not adopted in an effective manner (Zvolensky et al., 2020). The adopted theoretical stance argues that a greater sense of exposure helps individuals quantify risk in a more proactive manner to adopt preventive measures (i.e., vaccination; Carico et al., 2020). Hence, the current research proposed the following hypotheses.

H1: Perceived exposure influences the PHRs.

H2: PHR influences the health-protective behavioral intentions.

Zhou et al. (2021) stated that risk perception mediates the individuals’ judgment while mapping certain health risks, i.e., severity or susceptibility of exposure. They further emphasized that self-efficacy can play a strategic role in formulating susceptibility, severity, exposure, risk perception, and health-protective behavior. However, self-efficacy is less studied compared with the rest of the HBM’s constructs (Rosenstock et al., 1988; Zhou et al., 2021). MSE (Chen et al., 2020) and SSE (Yang et al., 2020) are also recorded as significant determinants to map an individual’s cognitive and behavioral change during COVID-19. Thus, the following hypothesis was proposed in the current research.

H3: (a and b): Perceived exposure influences an individual’s MSE and SSE.

Fundamentally, MSE comprises the ability to operate media (platform) and the attributes to access, understand, and interact with media (Hammer et al., 2021). Nur et al. (2016) argued that people know about the world through media. An individual’s confidence in accessing, analyzing, evaluating, interacting, and participating is related to media content. In the context of the current pandemic crisis, trust in media has been recorded as a significant construct that defines public health-protective behavior (Niu et al., 2021). Moreover, the source of information also helps define risk perception and related health-protective behavior (Wang et al., 2021). The challenge of MSE in the current pandemic is complex, because the impacts of international and domestic media sources vary in terms of the effect on society (Su et al., 2021). Therefore, the following hypothesis was proposed.

H4: MSE influences health-protective behavioral intentions.

SSE describes the individual’s ability to use scientific knowledge and understanding to assess and evaluate scientific information and arguments (National Research Council, 1996). Fasce and Picó (2019) argued that scientific thinking ability (efficacy) refers to the understanding of scientific theories, trust in science, and critical thinking. The literature argues that SSE can help an individual cope with cognitive anxiety, because it helps in the development of science engagement and is more effective in an informal setting (Yang et al., 2020). In the context of the COVID-19 pandemic crisis, the literature argues that greater belief in science helps an individual adopt positive health-protective behavior (i.e., wearing a mask; Stosic et al., 2021). Moreover, scientific communication with justification and multilingual support during COVID-19 can be considered as effective measures (Taragin-zeller et al., 2020). Thus, the following hypothesis was proposed in the current research.

H5: SSE influences health-protective behavioral intentions.

In health-related behavioral research, the perceived benefits of being protected have a significant impact on the definition of health-protective behavior (Wu et al., 2021). The benefits of being protected as a part of belief can positively influence the health-protective behavior (Becker et al., 1978). Almazyad et al. (2021) concluded that the benefits of being protected are the third most significant construct after perceived exposure and self-efficacy during COVID-19. Similar findings were recorded by Shahnazi et al. (2020), who showed the strategic role of benefits of being protected while mapping motivations to take preventive measures during COVID-19; benefits of being protected also help reduce the psychological barriers of being protective. The perceived barriers of being protected can be labeled as a subjective assessment of the costs or obstacles to the given health-protective behaviors (Didarloo et al., 2017). Barriers of being protected is a critical variable for forecasting health-protective behaviors in the existing literature. Jeong and Ham (2018) indicated that perceived barriers negatively influence the customers’ behavior. Similarly, Hu et al. (2017) stated that barriers of being protected negatively affect seasonal influenza vaccine acceptance. Interestingly, the studies emphasized that the role of barriers of being protected in the case of COVID-19 was recorded with mixed perceptions. For instance, mental health can be captioned as a double-edged sword in the pandemic; it can consistently be a barrier and a motivator to adopt health-protective measures, e.g., avoiding physical activities (Marashi et al., 2021). Zhao and An (2021) argued that barriers of being protected are weakly significant constructs to define health-protective behavior. Therefore, the following hypotheses are proposed in the current research setting.

H6: Benefits of being protected influences health-protective behavioral intentions.

H7: Barriers of being protected influences health-protective behavioral intentions.

Cues to action are among the least addressed and underdeveloped constructs in HBM (Champion and Skinner, 2008; Jones et al., 2015), and they demand more attention, because they can have internal (intrinsic) or externally (extrinsic) driven factors that can be manipulated (i.e., through biased reporting on social media) or are naturally occur (factual record). In the context of COVID-19, several researchers emphasized the role of cues while conceptualizing health-protective behavior (Walrave et al., 2020; Tsai et al., 2021). Literature also reported that cues to act in social media can trigger compromising circumstances for social and psychological well-being in society (Chao et al., 2020; Xue et al., 2021). Interestingly, in some cases, institutions and regulatory authorities’ cues are also significant to the implementation and practice of health-protective behavior (Al-Sabbagh et al., 2021). Hence, the current study proposed the following hypotheses by revisiting the role of “cues to act” in HBM and adapting Urban and Kujinga’s (2017) view of “regulatory and normative support.”

H8: Regulatory cues moderated the relationship between PHRs and health-protective behavioral intentions.

H9: Normative cues moderated the relationship between PHRs and health-protective behavioral intentions.

## Materials and Methods

### Measurement Scales

Conceptually, the designed quantitative survey comprised two subsections. The first subsection aimed to frame the demographic profiling of respondents and includes questions about the age, gender, education, most preferred source of information to find updates about COVID-19, and frequency of news received every day during the COVID-19 pandemic. In the second subsection, the constructs’ related items, specifically those adopted from the existing sources to avoid the instrumental reliability and validity issues, were listed. All constructs’ related items were scored using the seven Likert scales, in which a higher score indicated a higher degree of agreement with the statement. To ensure the reliability-related challenges, specifically in the case of Chinese natives, the designed instrument was back-translated, because the constructs were initially adopted from sources in English. The instrument was translated into Chinese and then reverse-translated to English with the help of different volunteer participants. Both English versions were compared and revised to deal with the challenge of instrumental validity, as suggested by Brislin (1970). Moreover, the instrument was revised to address language-, content-, and layout-related issues after a pretest. The revision was conducted by three faculty members from the School of Humanities and Social Sciences (University of Science and Technology of China) and two health experts to evaluate the relevancy and validity. The faculty members have expertise in behavioral mapping. The adopted questionnaire is listed in Appendices A and B.

### Collecting Data

To map the perceived health concerns and factors affecting health-protective behavior of sojourners and natives in China, an online quantitative survey was conducted during the second and third quarters of 2020 with the help of a digital survey platform WJX.^1^ This is a reliable data collection source in Mainland China (Sajjad et al., 2020). Specifically, the individuals who stayed in Mainland China during the timespan of the COVID-19 outbreak were taken as eligible respondents for the study. These included the natives and the sojourners (students, employees, or family-dependent individuals). The lucky draw of an amount ranging from RMB 1 to 5 RMB (RMB 1 = USD 0.14) was offered to the potential participants to speed up the data collection pace. The final version was distributed among more than 900 eligible individuals through WeChat. However, only 598 filled responses were received, whereas 163 response sets were excluded due to their incompleteness. The incomplete responses were the outcome of the data collection condition. Respondents were restricted from skipping questions. Therefore, incomplete responses can be the result of leaving the questionnaire resurvey in the pipeline. Non-response biases calculated by the Wilcoxon rank-sum tests helped gauge the difference between the collected sample responses during the second and third quarters of 2020. No significant difference was found among the subsets of the responses noted. The demographic profile of the collected sample is listed in Table 1.

**Table 1:** Demographic profile of respondents.

Characteristic		Chinese natives (241)	Sojourner in China (194)	Overall (435)
Gender	Male	153	137	290
	Female	88	57	145
Education	Attended Vocational School	27	-	27
	Attended School / College	161	38	199
	Attended University	53	156	209
Age	Under 25	32	13	45
	25–35	193	149	342
	Above 35	16	32	48
The preferred source of information in the case of COVID-19	Newspaper or Radio	08	-	08
	Television	77	-	77
	Internet (Web only)	43	98	141
	APP, i.e., Wibo, WeChat	61	73	134
	Friends circle	52	23	75
Frequency of COVID-19-related news read/received every day (news/incidents)	1–3	68	77	145
	4–5	84	63	147
	More than five	89	54	143

### Variation of Responses

The study comprised a single quantitative approach for behavioral modeling, as proposed in the study. To avoid instrument bias, Harman’s single-factor assessment was performed to measure the maximum variance among the proposed constructs, as suggested in the existing literature. Expressly, in the overall model, the maximum variance by single factor was 29.045. Furthermore, the common latent factor (CLF) measured as suggested by Song et al. (2019). The standard regression scores of a model with and without CLF were compared. However, no difference was recorded above 0.200. Therefore, the quantified results eliminated the issue of CMB in the research.

## Analysis

The research used structural equation modeling (SEM), where the variance-based approach was adopted. Notably, partial least-squares (PLS) was performed for hypotheses testing, as proposed in Section 3. The statistical tool “ADANCO v2.0.1” was used to compute the estimated and proposed models. PLS was performed, because (1) it helps test model fitness (2) it is preferred for estimating models, and (3) the existing pool of literature encourages the use of PLS for behavioral modeling. To examine the model fitness in further detail, the fitness indices from AMOS-SPSS were also taken into account. Specifically, measurement evaluation was performed on the overall model. However, the path analysis for each of the subgroups was examined in further detail during the structural path analysis.

### Measurement Evaluation

The explanatory factor analysis (EFA) was performed to examine the internal and external reliability of the constructs. In particular, the instruments’ Cronbach alpha (α), composite reliability (CR), Dijkstra–Henseler’s rho (ρ_A_), and average variance extracted (AVE) were computed. All scores obtained during the EFA were recorded above the least acceptable value, as shown in Table 2. Moreover, the satisfactory results were also computed in the case of each subgroup.

**Table 2:** Internal reliability testing for the OVERALL collected sample.

Construct	Items	Loadings	α	CR	AVE
Exposure	3	0.851–0.926	0.912	0.962	0.894
Perceived health risk	3	0.862–0.938	0.940	0.940	0.842
Media self-efficacy	3	0.864–0.917	0.925	0.944	0.857
Benefits of being protected	3	0.841–0.932	0.945	0.946	0.853
Scientific self-efficacy	3	0.824–0.904	0.935	0.935	0.877
Barriers of being protected	3	0.892–0.914	0.922	0.921	0.895
Health-protective behavior	3	0.847–0.931	0.953	0.943	0.872
Normative cues	4	0.867–0.907	0.944	0.913	0.938
Regulatory cues	4	0.812–0.918	0.937	0.947	0.849

The lower cutoff value for AVE is 0.5; least acceptable value for loadings, ρ_A_, Cronbach alpha is 0.7 as noted in Gelhard and von Delft (2016) and Hair et al. (2010).

Exp, Exposure; PHR, Perceived Health Risk; MSE, Media Self-Efficacy; SSE, Scientific Self-efficacy; BaBP, Barriers of Being Protected; BeBP, Benefits of Being Protected; NC, Normative cues; RQ, Regulatory cues; AVE, Average Variance Extracted; ρ_A_, Dijkstra-Henseler’s rho; α, Cronbach Alpha; CR, Composite Reliability.

Fornell and Larcker (1981) and Hetro and Monotrait ratio of correlation (HTMT) approach for the computation were used to measure the external reliability of the constructs. Notably, in Fornell and Larcker’s approach, the correlation scores were recommended to be lower than the AVE’s square root scores (Fornell and Larcker, 1981). For HTMT, the correlation score was advised and recorded below 0.90 (Henseler et al., 2014). The satisfactory discriminant reliability scores were recorded in the overall mode and in each subgroup (natives and sojourners in China). Thus, no traces of homological issues were observed while computing the external validity. The results are presented in tabular form in Appendix C Meanwhile, multicollinearity was tested by computing the variance inflation factor (VIF) for each item of the proposed constructs. All observed items’ VIFs were lower than the upper cutoff limit, as recommended by Petter et al. (2007). Moreover, the country-level multicollinearity was also recorded within the acceptable limits. Thus, no multicollinearity issue was recorded in the current research.

### Overall Fitness

The research measured the fitness of the proposed model by calculating the standardized residual scores (SRMR; i.e., its square root mean values) and unweighted discrepancy (i.e., its dULS-least squares and dG-geodesic scores). The smaller scores for standardized residual values signified the fitness of the proposed model (Henseler et al., 2016), as listed in Appendix D. Apart from PLS-based fitness scores from ADANCO, model fitness indices from AMOS-SPSS v24.0 were also considered to ensure the model fitness of the proposed model, as shown in Table 3. All fitness indices in the overall model and in each subgroup were recorded as significant, as suggested by Hu and Bentler (1999), except the NFI score in sojourners in China, and GFI scores in each subgroup. However, these three values were also supported and considered acceptable, as suggested by Belanger and Carter (2008). Thus, all fitness index scores from AMOS-SPSS supported the fitness of the proposed model.

**Table 3:** Model fitness recorded through AMOS-SPSS.

	Models	CMIN	df	CMIN/df	Non-Centrality		Relative		Absolute	
					CFI	RMSEA	TLI	NFI	GFI	AGFI
	Preferred cutoff			5.0^*^	≥0.950^*^	≤0.08^*^	≥0.950^*^	≥0.950^*^	≥0.900^*^	≥0.800^*^
1	Overall model	504.699	167	3.022	0.975	0.068	0.969	0.963	0.908	0.873
2	Natives in China	395.213	167	2.367	0.971	0.075	0.964	0.952	0.869	0.818
3	Sojourners in China	340.998	165	2.067	0.971	0.074	0.963	0.945	0.872	0.821

* Recommended limits by Hu and Bentler (1999).

### Hypotheses Testing

With the support of ADANCO v2.0.1, the structural model-related hypotheses were tested in the overall model and subsets (natives and sojourners in China), as proposed in Section 3. In the following subsection, the findings from the overall model and from the subgroup are discussed in detail.

#### Overall Model

In the overall model, all proposed hypotheses in the structural model were observed to be significant and supported except in the case of H7, where the perceived barriers of being protected were noted as nonsignificant. These observations were performed while mapping the overall model to define the health-protective behavior during the pandemic (COVID-19) in mainland China, as shown in Table 4. Moreover, the control variables (age and education) were noted as nonsupported. The total variance computed through the proposed constructs (*R*^2^) for MSE, PHR, SSE, and health-protective behavior for the overall model were 35.2, 40.3, 41.3, and 60.7%, respectively, in the overall model. The study computed the path significance (ρ-value) and coefficient scores (β) for the hypotheses declared in Section 3. The perceived exposure was recorded with a strong positive influence on the PHR (H1: *β* = 0.635, *ρ* < 0.05), and a similar trend was suggested by Shahnazi et al. (2020). The findings also concluded that PHR significantly affected the health-protective behavior (H2: *β* = 0.134, *ρ* < 0.05), and a similar trend was discussed by Carico et al. (2020). Moreover, perceived exposure was also observed as a significant influencer of the proposed efficacies, particularly the MSE (H3a: *β* = 0.593, *ρ* < 0.05) and SSE (H3b: *β* = 0.642, *ρ* < 0.05). These findings were aligned with the existing literature, in which efficacies helped formulate protective actions and responses to the pandemic crisis (Rui et al., 2021).

**Table 4:** Path analysis for overall model and subgroups (sojourners vs. natives).

Sr.	Hypotheses	Beta (regression scores)		
		Overall model	Natives in mainland China	Sojourners in mainland China
H1	Exp →PHR	0.635^***^	0.512^***^	0.730^***^
H2	PHR→HPB	0.134^*^	0.096^*^	0.175^**^
H3a	Exp→MSE	0.593^***^	0.557^***^	0.634^***^
H3b	Exp→SSE	0.642^***^	0.599^***^	0.706^***^
H4	MSE→HPB	0.276^***^	0.435^***^	0.202^*^
H5	SSE→HPB	0.214^**^	0.205^*^	0.175^*^
H6	BeBP→HPB	0.133^*^	0.067^*^	0.166^*^
H7	BaBP→HPB	0.022 ns	0.032 ns	0.041 ns
H8	RQ * PHR→HPB	0.090^***^	0.107^**^	0.060 ns
H9	NC * PHR→HPB	0.106^***^	0.069 ns	0.134^***^

Exp, Exposure; PHR, Perceived Health Risk; MSE, Media Self-Efficacy; SSE, Scientific Self-efficacy; BaBP, Barriers of Being Protected; BeBP, Benefits of Being Protected; NC, Normative cues; RQ, Regulatory cues.

* *p* < 0.05;

** *p* < 0.01;

*** *p* < 0.001.

MSE (H4: *β* = 0.276, *ρ* < 0.05) and SSE (H5: *β* = 0.214, *ρ* < 0.05) showed a significant impact on the definition of health-protective behavior, which was in line with the existing pool of literature (Niu et al., 2021; Stosic et al., 2021; Wang et al., 2021). Furthermore, the benefits of being protected were observed to be significant in the overall model (H6: *β* = 0.133, *ρ* < 0.05), as suggested by Al-Sabbagh et al. (2021) and Zampetakis and Melas (2021). However, the nonsignificant relationship between the perceived barriers of being protected and health-protective behavior (H7: *β* = 0.022, *ρ* > 0.05) highlighted the seriousness of the current pandemic crisis and the urgency to address the misaligned gap by improving effective communication with the citizens. Zhao and An (2021) also observed the weak relationship of the barriers of being protected with the health-protective behavior in a recent study.

The moderating effect in the H8 and H9 was computed with the help of the hierarchical regression model. The findings concluded that the regulatory cues (H8: *β* = 0.090, *ρ* < 0.05) and normative cues (H9: *β* = 0.106, *ρ* < 0.05) significantly affected the perceived health-protective behavior. A similar trend of findings was seen in the recent pool of literature, in which the government institutions were noted as significant determinants in defining public preventive behavior in China (Min et al., 2020). Moreover, social influence during the time of COVID-19 was identified as a strong predictor to define public consumer behavior, i.e., panic buying as a part of protective behavior (Naeem, 2021). The interaction plots used to visualize the moderating analysis are listed in Appendix D.

#### Natives vs. Sojourners in Mainland China

In the comparison of the natives and sojourners in China, brief psychological profiles of natives and sojourners were extracted from the current findings. All hypotheses in the proposed setting were significant, except for the role of barriers of being protected in the definition of individuals’ health-protective behavior in both cases (sojourner and natives). Moreover, education in the case of sojourners was noted as the only control variable that was recorded as significant. While defining PHR, the perceived fear of susceptibility and severity of COVID-19 in terms of exposure were found to have a greater effect on natives than on sojourners. The comparatively high concern of being exposed can be related to the panic among migrants (including sojourner; Ullah et al., 2021), as every responsible government is imposing safety measures to deal with COVID-19. The perceived exposure (concern) had a more significant impact on individuals’ media and SSE among sojourners than among natives. The current findings can be argued on the basis of the descriptive profile of the respondents, as most of the sojourners rely on digital media and attend academic institutions for their tertiary-level education. The graphical explanation of the proposed model in case of sojourners and natives is shown in Appendix D.

While mapping the exogenous factors to define perceived health-protective behavioral intentions of sojourners and natives in mainland China, the findings revealed that MSE is the strongest determinant in both cases. In-person communication has been replaced with digital medium and also drives a few challenges, i.e., COVID-19-related information overload (Song et al., 2021) and digital exclusion of nondigital natives and migrants (Nguyen et al., 2021). Thus, this finding signifies the sensitivity of MSE. Furthermore, the SSE was a slightly stronger determinant among natives than among sojourners. The findings are in line with those obtained in Flynn Murphy’s article in *Nature* (Murphy, 2020), where the capable medial workforce in China contributed to digital media as a scientific communicator to explain COVID-19 and communicate related health-protective initiatives with citizens. The PHRs from COVID-19 and perceived benefits of being protected were two-fold stronger determinants for defining perceived health-protective behavior among sojourners than among the native Chinese. This pattern of findings can potentially lead to a future study, in which the role of national culture can be taken into account. China, with its traditions and value of collectivism, was the first country to ask its citizens to wear masks, whereas the Western region was still confirming the effectiveness of mask-wearing practice by conducting clinical trials and research (Zurong, 2020); this information was intentionally ignored in the current research.

An interesting pattern can be obtained by analyzing the role of regulatory and normative cues to act among sojourners and natives. Specifically, regulatory support observed significant factor as a cue to act in the Chinese population, as suggested by Liu et al. (2020). However, sojourners mostly relied on cues from social networks (normative setting) only. Literature argues that more than 60% of the foreigners in China accessed COVID-19-related information through international sources (Chen and Liu, 2020), which can help portray sojourners’ nonsignificant absorption of regulatory cues. The interaction plots in case of moderation analysis are listed in Appendix D.

## Implications

The current research signifies the role of scientific and MSE and the crucial role of regulatory and normative cues while conceptualizing health-protective behavior of sojourners and natives in China. In terms of implications, the following theoretical and practical implications can be extracted from the current research.

The current research has several theoretical implications. First, the current research can distinguish results on the basis of its theoretical stance, as the proposed model for the study was adopted from HBM (a psycho-sociological model). It can predict and understand individuals’ health-protective behavioral intentions. Simultaneously, two primary pillars are adopted from IT, where the normative and regulative support are used to revisit the role of cues to act in the HBM: in particular, the adoption of regulative and normative support intended to address one of the least studied sections of HBM, as stated in the literature. Therefore, the study contributes its unique stance to the existing literature on HBM.

Second, besides the adaption of factors from IT, self-efficacy as a construct is also revisited. The current research proposed the critical role of media and SSE as determinants of perceived health-protective behavioral intentions during COVID-19. Theoretically, this revisiting of self-efficacy as a construct can be a part of a social cognitive theory or as an empirical research-based initiative to deepen the understanding and effectiveness of HBM. The current adopted view of self-efficacy can be suggested to examine health-protective behavioral intentions of digital natives or migrants or in the digital sphere of social setting.

Moreover, several research initiatives have studied the physical and psychological effects of outbreaks of severe infectious diseases according to different demographic variables, e.g., whether examining individuals from specific socio-economic settings or behavioral mapping in case of a specific segment of society on the basis of their demographic attributes. However, no studies have been performed on the physical and psychological impacts of outbreaks of COVID-19 on sojourners and natives. No research initiative ever tried to compare the efficacies or the impact of different cues to action among sojourners and natives in any particular setting.

Apart from the theoretical contributions of the current research, the findings of sojourners’ and natives’ health-related behavior during COVID-19 in the Mainland China have some practical implications that can be potentially generalized globally. First, the comparatively unequal effect of normative and regulatory support in case of sojourners and natives was recorded. The perceived effectiveness and sense of connectivity between institutions and individuals in dealing with pandemic crisis differed between natives and sojourners. The literature argues that migrants (including sojourners) usually feel more insecure and panicked during an infectious disease epidemic. Specifically, the nonsignificant impact of regulatory cues’ implies that the gap between institutions and individuals is comparatively high in the case of sojourners. Therefore, the findings imply that effective communication with sojourners can be a future area of concern for researchers, academicians, and policy makers, especially in China, where foreign-attractive reforms are in the early stage. For instance, one of the most constructive measures is the use of a multilingual or multimode communication mix, in which maximum interaction with sojourners becomes possible for institutions during the crisis. In terms of stakeholders’ management, the institution’s primary responsibility is to mobilize and involve each member of the society to make the crisis management strategy successful and effective.

Moreover, the theoretical implication to embed media and SSE in HBM also stretches the practical implications further. MSE was recorded as the most dominating construct in the overall model and in the case of natives and sojourners separately. Thus, MSE is a critical attribute in any scenario of a crisis control initiative. In recent years, the digital attributes of media allowed the creation of new perspectives of information-seeking and sharing behaviors in society, i.e., the pull architecture of the internet always brings much information to an information seeker. Furthermore, as discussed in Section 3, international and domestic media sources influence information seekers differently during the COVID-19 pandemic in mainland China. Therefore, the current study implies that the efficient and effective use of media by concerned institutions can help communicate information to sojourners and natives in a more digitally smart and effective manner i.e. the use of immersive media in risk communication. The study highlights the critical role of SSE, because it helps conceptualize individuals’ risk perception in any emergency crisis. The presence of a science museum in every populous city in China is a part of the national strategy to increase the public’s understanding of science and its impact on society. The significance of SSE implies that the use of science communication in a more informal manner during a pandemic is important. Lastly, sojourners are supposed to be ambassadors who can communicate with international entities. Therefore, the gap between the PHR and protective behavior must be minimized to avoid spreading international propaganda.

## Conclusion and Future Studies

The current research initiative was proposed to a persuasive psychological model to conceptualize the health-protective behavior of sojourners and natives in Mainland China during the COVID-19 pandemic crisis. The study made a theoretical contribution by addressing the role of media and SSE in health-protective behavioral intention modeling. Moreover, the role of regulatory and normative cues was also stretched further using the existing HBM. The possible list of future research fronts may include the following: (1) holistic communication strategy in a pandemic, especially in the presence of a communication barrier (i.e., multilingual support); (2) the role of efficacies need to be explored further, because they classify human cognitive abilities, i.e., how self-resilience and cognitive abilities help mitigate the pandemic situation; (3) the nonsignificant impact of regulative cues in sojourners highlights the need to improve sojourners’ understanding to distinguish the cues from institutions and society; (4) in terms of the public’s understanding of socio-scientific issues, the role of SSE should be emphasized and revisited; (5) the role of the trust in technology and in institutions, as well as the social capital in terms of individuals’ social networking, should be examined to map sojourners’ and natives’ health-protective behavioral intentions in routine life and under unique circumstances (i.e., pandemic situation).

## Data Availability Statement

The raw data supporting the conclusions of this article will be made available by the authors, without undue reservation.

## Author Contributions

FA and MA: conception and design of study. QY: acquisition of data. AS and MA: analysis and/or interpreting data. FK: drafting the manuscript. All persons who have made substantial contributions to the work reported in the manuscript. All authors contributed to the article and approved the submitted version.

### Conflict of Interest

The authors declare that the research was conducted in the absence of any commercial or financial relationships that could be construed as a potential conflict of interest.

## Footnotes

^1^www.wjx.cn
