# Supplementary material for: What Explains Natives and Sojourners Preventive Health Behavior in a Pandemic: Role of Media and Scientific Self-Efficacy
Source: Front Psychol. 2021 Jun 29;12:664399. doi: 10.3389/fpsyg.2021.664399 (PMC8275932; doi:10.3389/fpsyg.2021.664399)
Supplement: Supplementary file 1 [file Data_Sheet_1.docx]

**Appendix A**

**CHINESE VERSION**

**Perceived Susceptibility易感性认知** (Champion, 1999)

1可有可能我会感染上新型冠状病毒肺炎。

2我认为新型冠状病毒肺炎是严重的健康问题。

3我觉得我将感染新型冠状病毒肺炎在流行期间的某个时候。

**Benefits of Being protected感知被保护的益处** (Fallah Zavareh et al., 2018; Tajeri moghadam et al., 2020)

1我认为按照卫生专家的建议采取预防措施可以防止严重后果。

2在户外，通过采取安全措施(即社交距离和戴口罩)可以保护我远离新型冠状病毒肺炎。

3我相信，如果我遵循安全措施，我不会成为一个传染源对我周围的人。

**Science Self Efficacy科学自我效能感** (Nietfeld et al., 2006; White et al., 2019)

1.我相信我可以学习任何科学现象。

2.我很有自信在理解科学现象方面。

3.我有信息在在我开始倾听或参与任何科学问题的新话题之前，就能够理解它。

**Media Self Efficacy媒体自我效能感** (Chang et al., 2017)

我有信心利用现有的媒体获取信息(新闻)……

1如果我有指示可以参考的话。

2即使周围没有人教我怎么做。

3如果我以前使用过类似的信息源(新闻)系统。

**Perceived Health Risk** **健康威胁认知** (Yuen et al., 2020)

1如果我不采取安全措施，我接触新型冠状病毒肺炎的几率很高。

2如果我不执行安全行为，我将更有可能暴露于新型冠状病毒肺炎。

3一想到可能接触新型冠状病毒肺炎，我就非常害怕。

**Barriers of Being protected** **感知被保护的障碍** (Carico et al., 2020; Champion, 1999)

1我认为居家自我隔离和采取预防措施，使我远离社区。

2居家隔离给我带来了心理压力。

3采取预防措施下我很难获得日常必需品。

**Health Protective Behaviour** **健康防护行为** (Yuen et al., 2020)

1我将全力遵守所有安全规程(必要的或世界卫生组织建议的)。

2我遵守我认为必要的或世界卫生组织建议的安全规则。

3我尽我最大的努力获取每一个必要的信息来保护我自己。

**Normative Support Cues规范支持线索** (Tajeri moghadam et al., 2020)

在与我的朋友和家人交流时，我明白/获知….

1在处理当前紧急情况时必须采取的安全措施。

2在应对当前紧急情况时采取安全措施的益处。

3鼓励采取预防措施。

4戴上面具和保持社交距离，对保护自己有很大帮助。

**Regulatory Support Cues监管支持线索** (Tajeri moghadam et al., 2020)

相关政府部分积极地

1强调在应对新型冠状病毒肺炎时采取的安全防范措施是重要的。

2传播/沟通采取安全措施的益处(即通过公众意识运动)。

3使用所有类型的电子或印刷媒体来沟通关于新型冠状病毒肺炎相关的挑战

4努力控制/遏制新型冠状病毒肺炎的传播与扩散。

**Appendix B**

**ENGLISH VERSION**

**Perceived Susceptibility** (Champion, 1999)

1. It is likely that I will infected with COVID-19
2. I believe that COVID-19 is severe health problem
3. I feel I will get COVID-19 sometime during epidemic

**Benefits of Being protected** (Fallah Zavareh et al., 2018; Tajeri moghadam et al., 2020)

1. I believe that by taking precautionary measures as suggested by health experts can prevent from serious outcomes.
2. In the case of being outside home, by adopting safety measures (i.e. Social distancing and wearing mask) would protect me from COVID-19.
3. I believe that if I follow the safety measures I will not act like a carrier for other persons around me.

**Scientific Self Efficacy** (Nietfeld et al., 2006; White et al., 2019)

1. I am sure that I can learn about any scientific phenomena.
2. I have a lot of self-confidence when it comes to understand scientific phenomena.
3. Even before I begin to listen or get involved in any new topic in scientific issues, I feel confident I’ll be able to understand it.

**Media Self Efficacy** (Chang et al., 2017)

I am confident about using the available media to get information (news)….

1. … if I have instructions for reference.
2. … even if there is no one around to show me how to do it
3. … if I have used a similar source of information (news) system before.

**Perceived Health Risk** (Yuen et al., 2020)

1. My chance of getting exposed to COVID-19 are high if I do not perform safety behaviour.
2. I will be more likely be exposed to COVID-19 if I do not perform safety behaviour
3. The thought of being exposed to COVID-19 scares me.

**Barriers of Being protected** (Carico et al., 2020; Champion, 1999)

1. To stay at home in self-isolation and to take precautionary measures keeps me away from community I know.
2. Staying home is causing psychological pressure on me.
3. It’s very hard for me to get daily essentials with precautionary measures

**Health Protective Behaviour** (Yuen et al., 2020)

1. I follow all safety procedures (which are necessary or advised by WHO) at any cost.
2. I follow safety rules that I think are necessary or advised by WHO.
3. I tried my best to get every information necessary to protect myself.

**Normative Support Cues**  (Tajeri moghadam et al., 2020)

While communicating with my friends and family, I have heard about the….

1. … safety precautions which are important to be taken while dealing with current emergency situation.
2. … advantages of adopting safety measures while dealing with current emergency situation.
3. … the encouragement to adopt the precautionary measures.
4. … wearing mask and social distancing can help me a lot to protect myself.

**Regulatory Support Cues** (Tajeri moghadam et al., 2020)

Relevant government departments actively…

1. … emphasized that the safety precautions are important to be taken while dealing with COVID-19.
2. … communicated about the advantages of adopting safety measures (i.e. by public awareness campaigns).
3. … used all possible electronic or print media to communicate about COVID-19 related challenges.
4. … working to control the spread of COVID-19.

**Appendix C**

Abbreviation: Exp=Exposure, PHR=Perceived Health Risk, MSE=Media Self-Efficacy, SSE=Science Self-efficacy, BaBP=Barriers of Being Protected, BeBP= Benefits of Being Protected, NC=Normative cues, RQ=Regulatory cues

Table 3: External Validity check: Fornell-Lacker for OVERALL collected sample.

| Construct | Exp | PHR | MSE | BeBP | SSE | BaBP | HPB | NC | RQ |
| --- | --- | --- | --- | --- | --- | --- | --- | --- | --- |
| Exp | .945 |  |  |  |  |  |  |  |  |
| PHR | .403 | .971 |  |  |  |  |  |  |  |
| MSE | .351 | .399 | .978 |  |  |  |  |  |  |
| BeBP | .384 | .433 | .440 | .924 |  |  |  |  |  |
| SSE | .412 | .429 | .392 | .383 | .936 |  |  |  |  |
| BaBP | .467 | .429 | .455 | .402 | .485 | .946 |  |  |  |
| HPB | .319 | .407 | .471 | .411 | .435 | .438 | .934 |  |  |
| NC | .325 | .251 | .430 | .318 | .230 | .383 | .228 | .969 |  |
| RQ | .443 | .478 | .659 | .511 | .578 | .558 | .557 | .363 | .921 |

| Construct | Exp | PHR | MSE | BeBP | SSE | BaBP | HPB | NC | RQ |
| --- | --- | --- | --- | --- | --- | --- | --- | --- | --- |
| Exp |  |  |  |  |  |  |  |  |  |
| PHR | .635 |  |  |  |  |  |  |  |  |
| MSE | .592 | .632 |  |  |  |  |  |  |  |
| BeBP | .620 | .659 | .664 |  |  |  |  |  |  |
| SSE | .642 | .655 | .626 | .620 |  |  |  |  |  |
| BaBP | .684 | .655 | .675 | .635 | .697 |  |  |  |  |
| HPB | .565 | .638 | .686 | .642 | .659 | .661 |  |  |  |
| NC | .570 | .501 | .656 | .564 | .480 | .619 | .477 |  |  |
| RQ | .665 | .691 | .812 | .715 | .761 | .746 | .746 | .602 |  |

Table 4:External Validity check: HTMT for OVERALL collected sample.

Table 5: External Validity check: Fornell-Lacker (RESPONDENTS: NATIVE in MAINLAND CHINA).

| Construct | Exp | PHR | MSE | BeBP | SSE | BaBP | HPB | NC | RQ |
| --- | --- | --- | --- | --- | --- | --- | --- | --- | --- |
| Exposure | .953 |  |  |  |  |  |  |  |  |
| PHR | .532 | .975 |  |  |  |  |  |  |  |
| MSE | .401 | .398 | .985 |  |  |  |  |  |  |
| BeBP | .500 | .502 | .376 | .905 |  |  |  |  |  |
| SSE | .498 | .489 | .359 | .393 | .957 |  |  |  |  |
| BaBP | .515 | .426 | .460 | .384 | .499 | .932 |  |  |  |
| HPB | .377 | .469 | .405 | .408 | .410 | .400 | .939 |  |  |
| NC | .316 | .280 | .527 | .398 | .225 | .482 | .261 | .982 |  |
| RQ | .598 | .564 | .536 | .469 | .670 | .628 | .515 | .431 | .916 |

Table 6: External Validity check: HTMT (RESPONDENTS: NATIVE in MAINLAND CHINA).

| Construct | Exp | PHR | MSE | BeBP | SSE | BaBP | HPB | NC | RQ |
| --- | --- | --- | --- | --- | --- | --- | --- | --- | --- |
| Exp |  |  |  |  |  |  |  |  |  |
| PHR | .730 |  |  |  |  |  |  |  |  |
| MSE | .633 | .631 |  |  |  |  |  |  |  |
| BeBP | .709 | .709 | .615 |  |  |  |  |  |  |
| SSE | .706 | .699 | .599 | .628 |  |  |  |  |  |
| BaBP | .717 | .653 | .679 | .620 | .707 |  |  |  |  |
| HPB | .614 | .685 | .636 | .640 | .640 | .631 |  |  |  |
| NC | .562 | .529 | .726 | .632 | .474 | .695 | .510 |  |  |
| RQ | .773 | .752 | .732 | .687 | .819 | .792 | .717 | .657 |  |

Table 7: External Validity check: Fornell-Lacker (RESPONDENTS: SOJOURNERS IN MAINLAND CHINA).

| Construct | Exp | PHR | MSE | BeBP | SSE | BaBP | HPB | NC | RQ |
| --- | --- | --- | --- | --- | --- | --- | --- | --- | --- |
| Exp | .928 |  |  |  |  |  |  |  |  |
| PHR | .262 | .965 |  |  |  |  |  |  |  |
| MSE | .310 | .404 | .970 |  |  |  |  |  |  |
| BeBP | .279 | .364 | .525 | .950 |  |  |  |  |  |
| SSE | .359 | .371 | .443 | .373 | .911 |  |  |  |  |
| BaBP | .435 | .440 | .448 | .434 | .465 | .969 |  |  |  |
| HPB | .277 | .340 | .575 | .418 | .466 | .501 | .926 |  |  |
| NC | .438 | .226 | .310 | .226 | .231 | .247 | .177 | .947 |  |
| RQ | .284 | .387 | .835 | .557 | .477 | .475 | .619 | .287 | .933 |

Table 8: External Validity check: HTMT (RESPONDENTS: SOJOURNERS IN MAINLAND CHINA).

| Construct | Exp | PHR | MSE | BeBP | SSE | BaBP | HPB | NC | RQ |
| --- | --- | --- | --- | --- | --- | --- | --- | --- | --- |
| Exp |  |  |  |  |  |  |  |  |  |
| PHR | .512 |  |  |  |  |  |  |  |  |
| MSE | .557 | .635 |  |  |  |  |  |  |  |
| BeBP | .528 | .603 | .726 |  |  |  |  |  |  |
| SSE | .599 | .609 | .665 | .611 |  |  |  |  |  |
| BaBP | .659 | .663 | .669 | .659 | .685 |  |  |  |  |
| HPB | .526 | .583 | .758 | .646 | .683 | .707 |  |  |  |
| NC | .662 | .475 | .557 | .477 | .481 | .498 | .421 |  |  |
| RQ | .533 | .622 | .614 | .748 | .691 | .689 | .787 | .536 |  |

**Appendix D**

Table 9: Structural (Goodness measures) computed from ADANCO.

| **Model** | **Discrepancy** | **Score** | **HI95** | **Result** |
| --- | --- | --- | --- | --- |
| Overall Model | SRMR | .026 | .247 | Supportive |
|  | d_ULS_ | 2.071 | 24.383 | Supportive |
|  | d_G_ | 1.377 | 9.427 | Supportive |

Figure 1: Comparative analysis between sojourners and natives Health protective behaviour in Mainland China.

1. Regulatory (b) Normative

Figure 2: Overall (a) Regulatory and (b) Normative support's moderation interaction in case of PHR and HPB.

1. Regulatory (b) Normative

Figure 3: Natives' (a) Regulatory and (b) Normative support's moderation interaction in case of PHR and HPB.

.

1. Regulatory (b) Normative

Figure 4: Sojourners’ (a) Regulatory and (b) Normative support's moderation interaction in case of PHR and HPB.

References

Bruns, D.P., Kraguljac, N.V., Bruns, T.R., 2020. COVID-19:Facts,Cultural Considerations,and Risk of Stigmatization. J. Transcult. Nurs. 00, 1–7. https://doi.org/10.1177/1043659620917724

Carico, R. “Ron,” Sheppard, J., Thomas, C.B., 2020. Community pharmacists and communication in the time of COVID-19: Applying the health belief model. Res. Soc. Adm. Pharm. 1–4. https://doi.org/https://doi.org/10.1016/j.sapharm.2020.03.017

Champion, V.L., 1999. Revised susceptibility, benefits, and barriers scale for mammography screening. Res. Nurs. Health 22, 341–348. https://doi.org/10.1002/(sici)1098-240x(199908)22:4<341::aid-nur8>3.3.co;2-g

Chang, H.H., Wong, K.H., Li, S.Y., 2017. Applying push-pull-mooring to investigate channel switching behaviors: M-shopping self-efficacy and switching costs as moderators. Electron. Commer. Res. Appl. 24, 50–67. https://doi.org/10.1016/j.elerap.2017.06.002

Fallah Zavareh, M., Mohamadi Hezaveh, A., Nordfjærn, T., 2018. Intention to use bicycle helmet as explained by the Health Belief Model, comparative optimism and risk perception in an Iranian sample. Transp. Res. Part F Traffic Psychol. Behav. 54, 248–263. https://doi.org/10.1016/j.trf.2018.02.003

Henseler, J., 2017. ADANCO 2.0.1 : User Manual. Kleve, Germany.

Nietfeld, J.L., Cao, L., Osborne, J.W., 2006. The effect of distributed monitoring exercises and feedback on performance, monitoring accuracy, and self-efficacy. Metacognition Learn. 1, 159–179. https://doi.org/10.1007/s10409-006-9595-6

Tajeri, M., Raheli, H., Zarifian, S., Yazdanpanah, M., 2020. The power of the health belief model ( HBM ) to predict water demand management : A case study of farmers ’ water conservation in Iran. J. Environ. Manage. 263, 110388. https://doi.org/10.1016/j.jenvman.2020.110388

Tajeri moghadam, M., Raheli, H., Zarifian, S., Yazdanpanah, M., 2020. The power of the health belief model (HBM) to predict water demand management: A case study of farmers’ water conservation in Iran. J. Environ. Manage. 263, 110388. https://doi.org/10.1016/j.jenvman.2020.110388

White, A.M., DeCuir-Gunby, J.T., Kim, S., 2019. A mixed methods exploration of the relationships between the racial identity, science identity, science self-efficacy, and science achievement of African American students at HBCUs. Contemp. Educ. Psychol. 57, 54–71. https://doi.org/10.1016/j.cedpsych.2018.11.006

Yuen, K.F., Li, K.X., Ma, F., Wang, X., 2020. The effect of emotional appeal on seafarers’ safety behaviour: An extended health belief model. J. Transp. Heal. 16, 100810. https://doi.org/10.1016/j.jth.2019.100810

Zhou, R., Wang, D., Siddiquei, A.N., Anwar, M.A., Hammad, A., Asmi, F., Ye, Q., Nawaz, M.A., 2019. GMO/GMF on Social Media in China: Jagged Landscape of Information Seeking and Sharing Behavior through a Valence View. Int. J. Environ. Res. Public Health. https://doi.org/10.3390/ijerph16234838
